# Probing the potentiality of the defoliator *Cricula trifenestrata* Helfer silk: a revisit

**DOI:** 10.1186/s42269-021-00669-w

**Published:** 2021-12-11

**Authors:** Hemachandran Hridya, Lopamudra Guha, Mahashankar Mazumdar, B. N. Sarkar, Soni Vijayakumar, P. Borpuzari

**Affiliations:** 1 Muga Eri Silkworm Seed Organisation, Ministry of Textiles, Central Silk Board, Guwahati, Assam India; 2 MESSO, P4 Seed Station, Mendipathar, Central Silk Board, North Garo Hills, Meghalaya India; 3 Regional Sericultural Textile Research Station, Central Silk Board, Guwahati, Assam India

**Keywords:** Wild, Silkworm, Cocoon, *Cricula*, Pest, Product, Silk

## Abstract

**Background:**

Transformation of pest to valuable product is considered to be a noteworthy innovation. This article explores the potentiality of wild silkworm *Cricula trifenestrata* Helfer for sustainable development towards human livelihoods.

**Results:**

The innate characteristics of this silkworm with robust rearing capacity have bestowed various aspects of biomaterials with special context to diversification of wild silk products. Views on challenges, prospects and the enigma of converting a pest to beneficial product are also unraveled. Exploration on utmost utilization of raw silk, scope for varied byproduct from silk waste may contribute a ray of hope for income generation to the rural population.

**Conclusion:**

With suitable plantation and congenial climatic conditions for rearing *Cricula trifenestrata* may serve as an alternative wild silk in contributing to the country’s wild raw silk production.

## Background

Wild/Vanya silkworm confers to non-mulberry silkworm which comprises *Antheraea mylitta, Antheraea proylei, Antheraea assamensis* and *Samia ricini* which are commercially exploited in India. In other countries wild silkworms which are commercially exploited includes *Gonometa, Hylaphora*, *Antheraea yamamai, Antheraea pernyi* etc. In the present scenario, India’s vanya raw silk production is around 10,581 MT (29.5%) against the country’s total production of 35,820 MT for the year 2019–20. In 2020–21, despite of covid-19 pandemic 33,739 MT of Raw silk was produced in which 9879 MT (29.2%) is contributed by vanya silk (Source Central Silk Board, Bangalore, India). There are numerous wild silkworm in India, which are least concerned for its probable productivity. An attempt is made to discuss about a common pest which can be transformed into a commercial product with scientific and technological intervention.

**Fig. 1:**
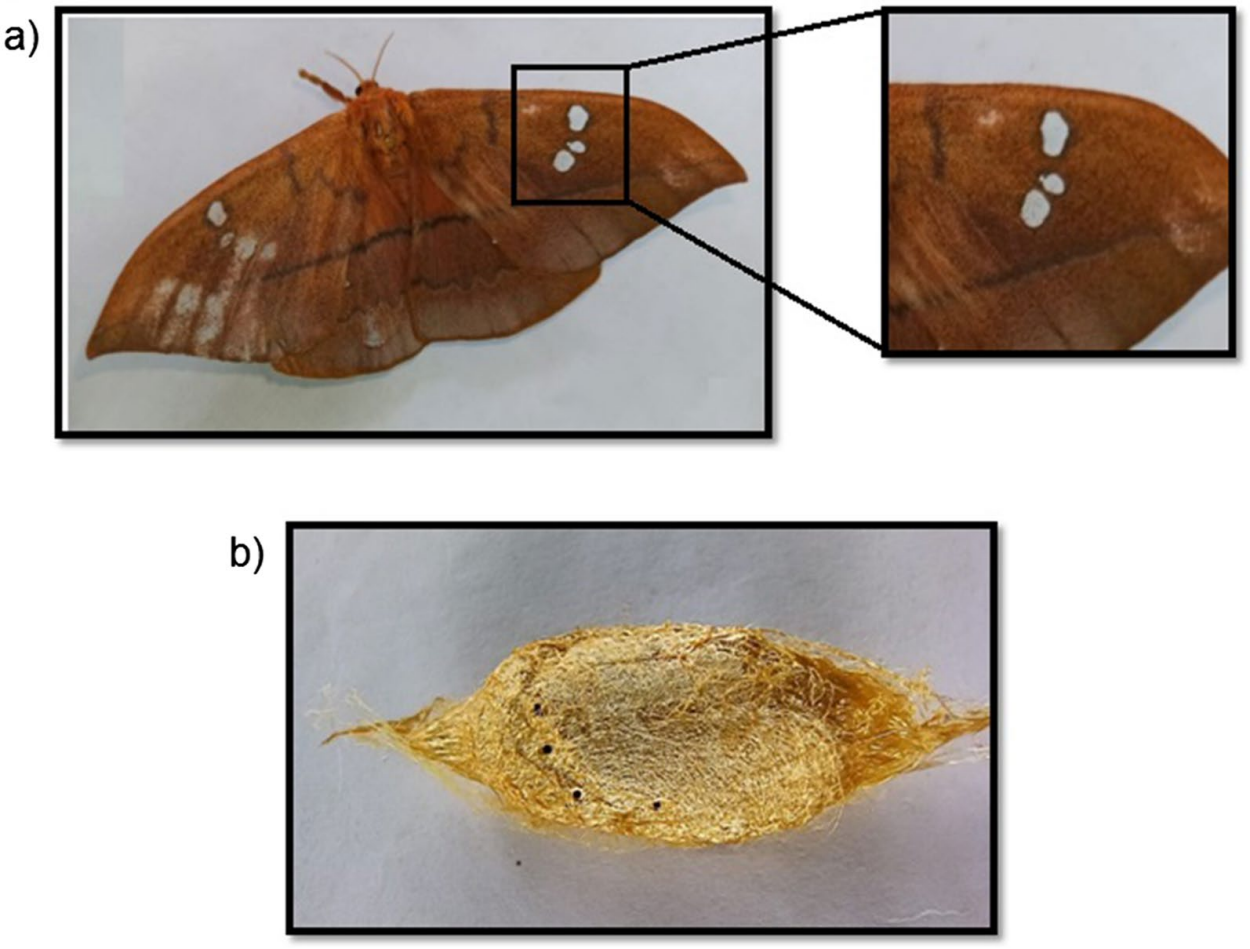
*Cricula trifenestrata* moth and Cocoon

**Fig. 2:**
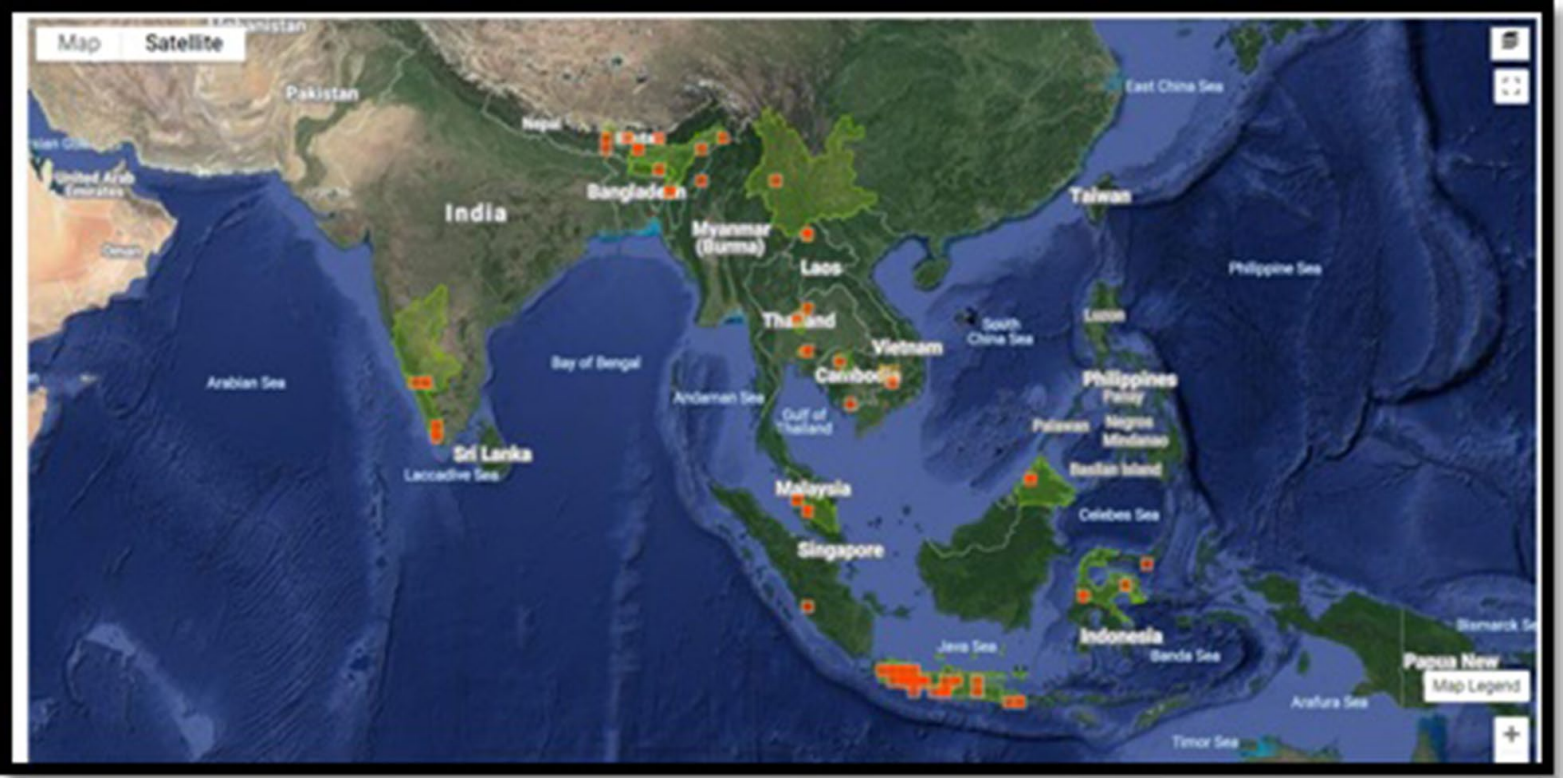
Global distribution of *Cricula trifenestrata*. *Source:* https://www.inaturalist.org/taxa/510228-Cricula-trifenestrata

*Cricula trifenestrata* Helfer is a wild Lepidopteron sericigenous moth known for its lustrous golden cocoon. The Genus *Cricula* was proposed by Walker in 1855. Capt. Jenkins and Helfer coined the species trifenestrata due to the presence of 3 windows like structure in the forewing as represented in Fig. 1 (Tikader et al. 2014; Kaleka et al. 2018). It is invariably considered as a pest for *Antherea assamensis* i.e., muga silkworm as it depletes the foliage by feeding thereby affecting the Effective Rearing Rate (ERR) of Muga silkworm. They infect a wide range of host plants namely *Mangifere indica, Persea bomycina, Anacardium occcidentale, Arachis hypogeal Cinnamomum cassia, litchi cheinensis, Machilus odoratissima* and *Camellia sinensis* (Ahmed et al 2012; Gharde and Chaudhuri 2018; Narang and Gupta 1979; Tikader et al. 2014). Host diversity of *Cricula trifenestrata* is detailed in (Table 1). *Cricula trifenestarata* is widely distributed in South Asian countries (Fig. 2). *Cricula trifenestrata* is reported to be predominant in India, Andaman, Myanmar, Vietnam, Cambodia, Malaysia, Singapore, Thailand, Bangladesh, Java, Philippines countries (Tikader et al. 2014; UK 2014).

**Table 1:** Host plant diversification of *Cricula trifenestrata*

S. no	Botanical name	Family	Common name
1.	*Mangifera indica*	Anacardiaceae	Mango
2.	*Persea bombycina /Machilus bombycina*	Lauraceae	Som Sum, Mojali, Majti
3.	*Anacardium occcidentale L*	Anacardiaceae	Cashew, caju
4.	*Arachis hypogeal L*	Fabaceae	Peanut, Groundnut, earthnut, monkey nut
5.	*Cinnamomum cassia*	Lauraceae	Chinese cassia, Chinese cinnamomum
6.	*Litchi cheinensis*	Sapindaceae	Lychee, litchi
7.	*Camellia sinensis*	Theaceae	Tea
8.	*Amomum subulatum*	Zingiberaceae	Black cardamom, Big cardamomum, hill cardamomum, winged cardmomum
9.	*Acrocarpus fraxinifolius*	Fabaceae	Pink cedar, Shingle tree, Australian Ash, Indian Ash, Red cedar, Keny coffeeshade
10.	*Bucklandia populnea*	Hamamelidaceae	Dingdah, Pipli, Singliang
11.	*Careya arborea*	Lecythidaceae	Patana Oak, Wild Guava
12.	*Schleichera trijuga*	Sapindaceae	Lac tree, Ceylon oak, Kusum tree
13.	*Ziziphus jujube*	Rhamnaceae	Jujube, red date, Chinese date, Chinese jujube Bogari
14.	*Piper nigrum*	Piperaceae	Black pepper, kalimirch, golmirch
15.	*Machilus odoratissima*	Lauraceae	Fragrant Bay,
16.	*Persea Americana*	Lauraceae	Avacado
17.	*Mimusops elengi*	Sapotaceae	Spanish cherry, bullet wood, Maulsari, Bakull, Elengi
18.	*Abelmoschus esculentus*	Malvaceae	Okra, Ladys’ finger
19.	*Altingia excelsa*	Hamamelidaceae	Rasamala, Oriental Sweet Gum, Shilarasa, Turushka
20.	*Cinchona officinalis*	Rubiaceae	Peruvian bark, quinine
21.	*Cinnamomum verum*	Lauraceae	Ceylon cinnamon, Dalchini, true cinnamon, lavanga
22.	*Elaeocarpus floribundus Blume*	Elaocarpaceae	Indian olive, Jalpai
23.	*Litsea glutinosa*	Lauraceae	Soft bolly gum, bolly beech, brown beech
24.	*Luffa aegyptiaca*	Cucurbitaceae	Sponge gourd, Ghia torai, loofa
25.	*Ziziphus mauritiana*	Rhamnaceae	Indian plum, Ber, elandai
26.	*Syzygium cumini (L)*	Myrtaceae	Java plum, jamun, black plum, naagai
27.	*Spondias sps*	Anacardiaceae	Hog Plum
28.	*Bischofia trifoliata*	Euphorbiaceae	Bishop wood
29.	*Canarium harveyi*	Burceraceae	Java almond, pili nut, pacific almond
30.	*Malus floribunda* Siebold ex Vanhoutte	Rosaceae	Crab apple, chokeberry

**Fig. 3:**
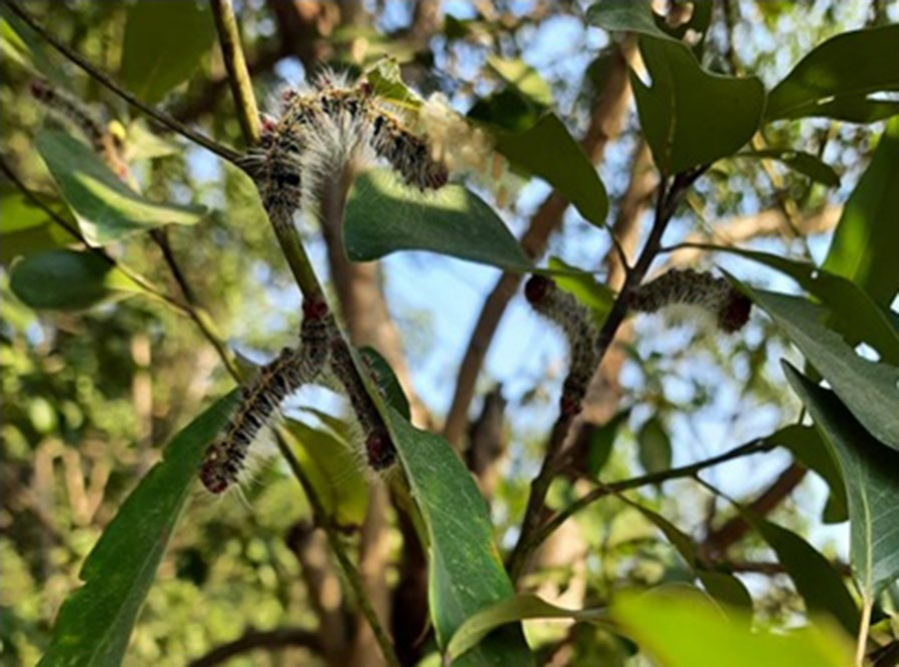
*Cricula trifenestrata* infestation on *Persea bombycina* foliage

**Fig. 4:**
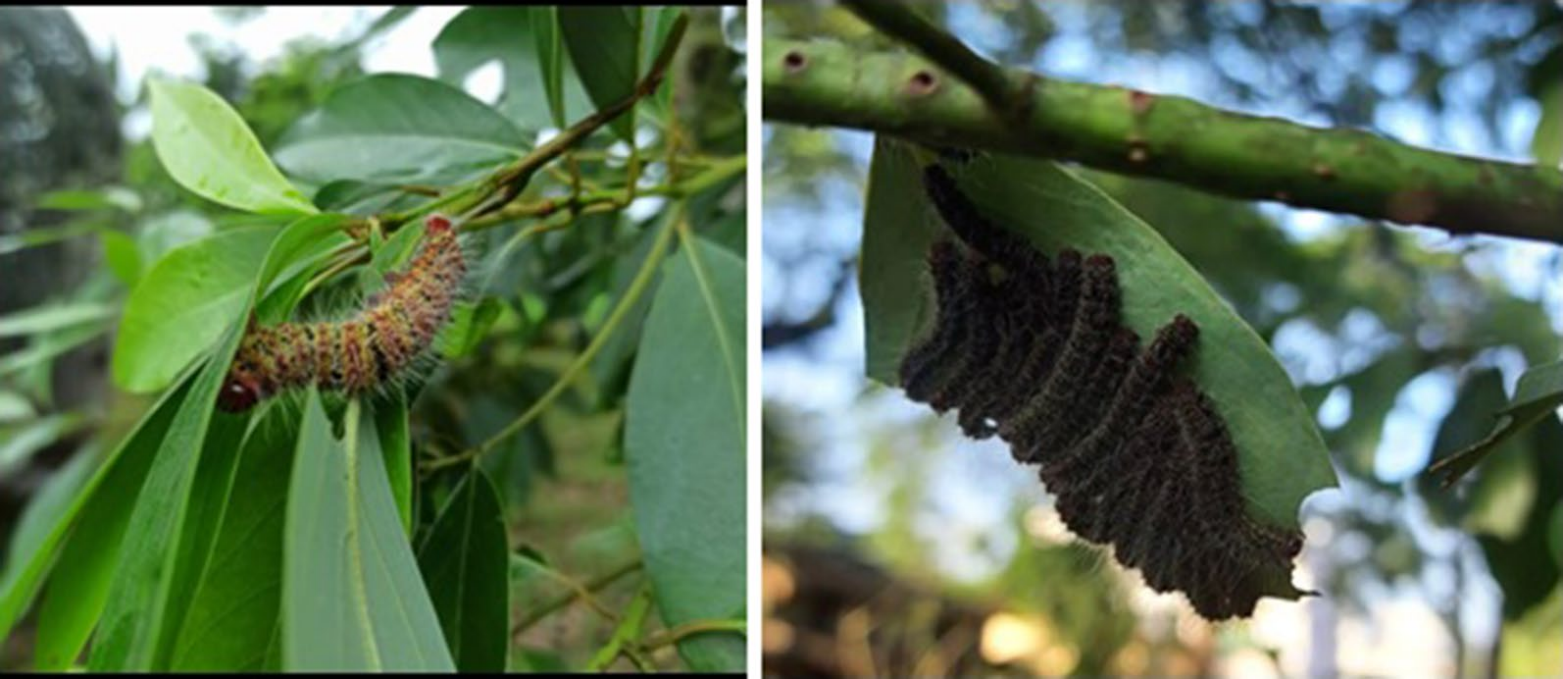
Different stages of *Cricula trifenestrata* larval infestation in *Persea bombycina* host

*Cricula trifenestrata* takes up to 61–125 days to complete the life cycle. Complete defoliation occurs in 4th and 5th instar. The larvae have 5 instars and passes 4 generation in a year (Tikader 2012; Huq et al. 1991). The average fecundity was reported to be 141.7 ± 11.79, and observed hatching rate was 88.81 ± 1.37% when reared in *Mangifera indica*. The average *Mangifera* leaf consumption was about 29.4 g (Amin et al. 2008) *Persea bombycina* fed larvae (Figs. 3, 4) exhibited higher content of protein and lipid in the haemolymph suggesting *Persea bombycina* as the superior host in respect to silk deposition (Ghosh et al. 2015). High Infestation in *Persea bombycina* was observed during September to January (Ahmed et al. 2012). The efficiency of conversion of digested food in first instar when fed with *Persea bombycina* was 36.54% and the digestability was 67.74% indicating *Persea bombycina* as the preferred host plant by *Cricula* on the basis of nutritional indices. (Biswas et al. 2013). *Cricula trifenestrata* were even recorded, at an altitude of 1097 m in some parts of Tamilnadu (Singh 1992). In cashew, *Cricula trifenestrata* larval infestation had a positive correlation with number of flower and seeds (Siswanti et al. 2017).

In Sikkim, *Cricula* was reported infesting Cardamom i.e., *Amomum subulatum* causing huge devastation of the crop. The average fecundity was observed to be 157–252 eggs per month. The mature larvae is black-brown with red sublateral stripes, 2–11 somites with six setiferous tuburcles. Larval period ranges to 87.16 + 2.65 days and larval mortality 23.23%. Life cycle takes around 168.92 days (Yadav and Kumar 2004). The chromosome number (*n*) of *C. trifenestrata* was found to be 31 (Narang and Gupta 1979). The cytochrome C oxidase subunit gene of *Cricula trifenestrata* was studied to identify specific unique sites for species identification to develop species marker. It was noted that 595 nucleotides are conserved in the species of cytochrome C oxidase gene. The 107th amino acid (valine) and 138th (threonine) were diagnostics amino acid for *C. trifenestrata*. The phylogentic study revealed that *Cricula* and *Antherea* share the same node of the saturnidae family suggesting monophyletic (Solihin et al. 2012).

### Control measures

The *Cricula* infestation causes havoc destruction to the foliage hence researchers have studied various physical, chemical and biological control methods to prevent infestation in field crops. Proper weeding of the tree cover in summer is seen effective to prevent to *Cricula* larval infestation in Cinchona plantations. This cultural operation serves as a first line of physical defence in preventing infestation (Van Zwet 1950). Biological control by utilizing parasitoid serves as an effective control measure in controlling the *Cricula* (Table 2). Parasitizaton o*f Cricula trifenestrata* eggs with *Telenomus* sp has complete efficacy up to pupal stages of. *Cricula trifenestrata,* when parasitized with *Brachymeria criculae* efficacy resulted in *79.71%. Mesomys orientalis* and *Sarcophaga sp.* parasitoid can also control the *Cricula* infestation during pupal stage (Ali and Karim 1991). *Xanthopimpla konowi* parasitizes the pupae (Singh 1992). *Brachymeria tibialis* (Walker) parasitoid destroys and consumes the pupae inside the cocoon preventing future multiplication. Hence *Brachymeria tibialis* can be efficiently utilized as a biological control in *Antherea assamensis* silkworm rearing (Tikader 2012).

**Table 2:** Reported Biological control of *Cricula trifenestrata* at different stages of rearing

S. no	Parasitoid	Developmental stage	Efficiency	References
1.	*Telenomus* sp	Pupae/egg	100%	Ali and Karim (1991)
2.	*Brachymeria criculae*	Pupae	*79.71%*	
3.	*Mesomys orientalis*	Pupae	Not defined	
4.	*Sarcophaga sp*	Pupae		
5.	*Brachymeria tibialis*	Pupae	25%	Tikader (2012)
6.	*Chalcis cricula*	larvae	Not defined	Yadav and Kumar (2004)
7.	*Chalcis etiplaeae*	larvae		
8.	*Xanthopimpla konowi*	larvae		Singh (1992)
9.	*Beauveria bassiana*	moth	Spore suspension 5 × 10^6^	Sjafaruddin and Rahmatia (1999)
10.	*Blepharipa zebina*	larvae	Not defined	Negi et al. (1993)
11.	*Exorista sorbillans*	pupae	13–14%	Sarma et al. (2006)
12.	*Pediobius* sp	pupae	66.4%	Alam and Ahmed (1992)

In Indonesia, *Beauveria bassiana* at a concentration of 5 × 10^6^ spores suspension resulted in highest mortality of *Cricula* moth. However it is not effective in *Antherea assamensis* field (Sjafaruddin and Rahmatia 1999). Parasitoid *Blepharipa zebina* infest larvae of *Cricula trifenestrata* which infest *Machilus bombycina* (Negi et al. 1993). In Arunachal Pradesh *Exorista sorbillans* was observed as an endoparasitoid parasitizing the *Cricula* larvae and causing death at pupal stage during March to mid-September (Sarma et al. 2006). *Chalcis cricula* and *C. etiplaeae* parasitises *Cricula* larvae (Yadav and Kumar 2004).

Fenvalerate and cypermethrin were the most effective chemical control methods, when treated for 7 days post treatment resulted in 94.44 and 79.16% larval mortality (Munaan 1986). In Maharastra field study has resulted in effective control of *Cricula* infestation in ground nut by cyhalothrin and Naled at 0.0005 and 0.001% (Deshmukh 1992) chemically infestation can be controlled by endosulphan, deltamethrin and neem based pesticide like azadirachtin (0.003% and 0.015%) has been effective (Ahmed et al. 2012) 0.05% endosulfan and parathion-methyl was used to control (Singh 1992). 0.1% methyl parathion and endosulphan are effective chemical control methods reported (Yadav and Kumar 2004).

## Methods

The rearing parameters are reported in varied host plants. Here attempts on exploration of post cocoon parameters are explored for commercial utilization. Cocoons of *Cricula trifenestrata* which was reared upon consuming *Persea bombycinia* in the wild were obtained and the pupal and shell weight was determined. The cocoons were stifled in hot air oven at 45 °C for 2 h. Owing to the presence of perforations in the cocoons reeling isa constrain hence it is utilized for spun yarn.

### Degumming and cake preparation

The removal of sericin and letting the fibres loose for further processing is called Degumming. Cocoons of *Cricula trifenestrata* were degummed by boiling in 0.5% sodium carbonate for 45 min. Further rinsing in hot distilled water and distilled cold water. Drying was done in hot air oven at 45 °C for 2 h.

$${\text{Degumming}}\,{\text{loss}}\,{\text{(\%)}} = W_{1} - W_{2} /W_{1}$$

where *W*_1_ is the initial weight of conditioned *Cricula* cocoons samples and *W*_2_ is the weight of degummed as well as conditioned *Cricula* cocoons sample Followed by washing. The individual degummed cocoons were opened in water and sheets are formed and placed overlapping. Cakes were prepared by overlapping 3–4 cocoon fibre sheets and dried in room temperature (Munshi et al. 2016).

### Spinning

The yarn is spin in a motorized spinning machine. The silk ratio and silk recovery is determined.

## Results

**Fig. 5:**
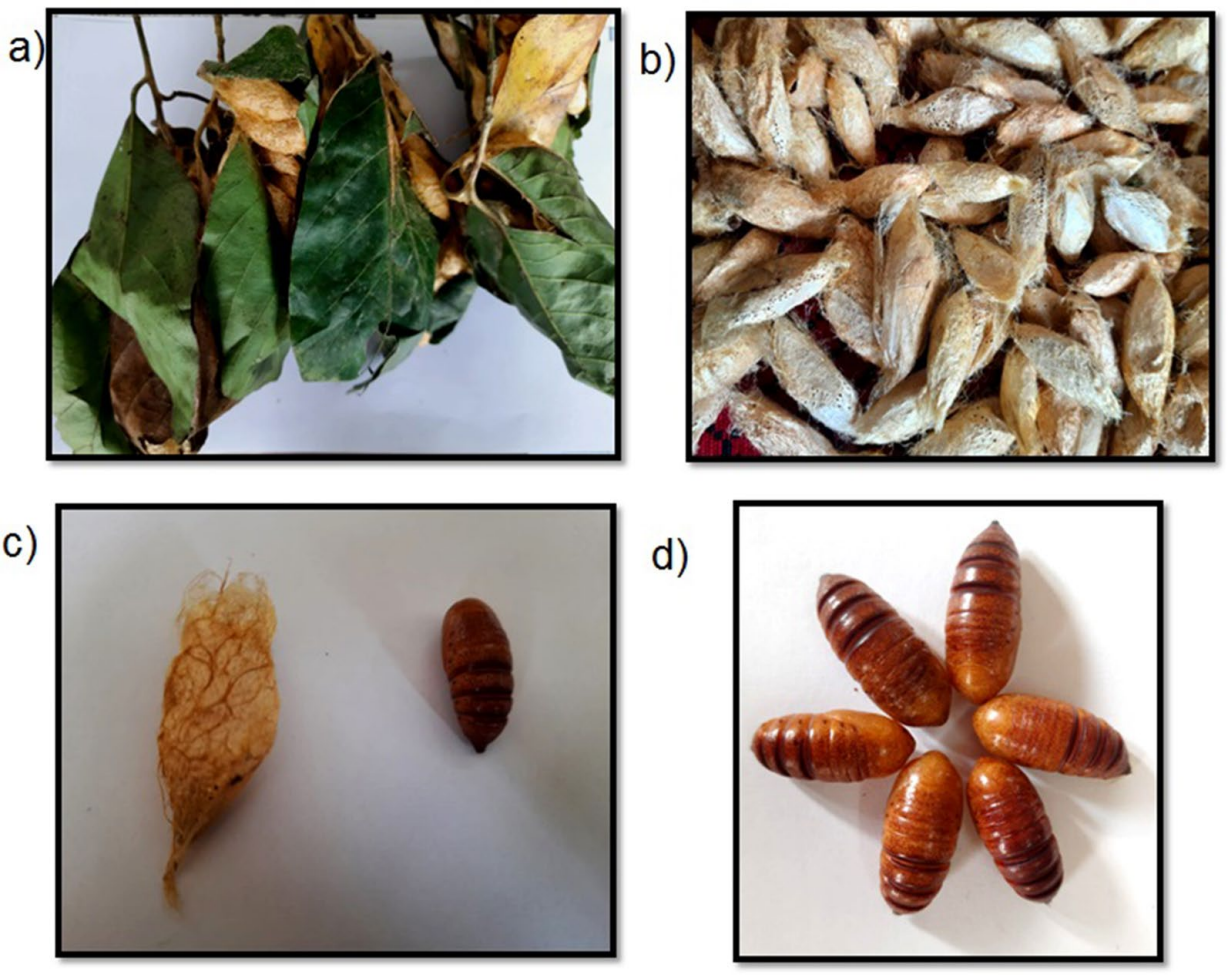
**a** Spinned cocoons in leaves, **b** collected cocoons reared on *Persea bombycina*, **c** single cocoon and pupae, **d** matured pupa

145 cocoons were obtained from the wild (Fig. 5). The post cocoon parameters were observed and analyzed and the results are shown in Table 3.

**Table 3:** Post cocoon parameters determined in *Cricula trifenestrata* cocoon

S. no	Post cocoon parameters assessed	Results in gm/%
1.	No. of cocoon	145
2.	Total Cocoon weight	105 gm
3.	Total shell weight	15.5 gm
4.	Total pupal weight	89.5 gm
5.	Single cocoon weight	0.72 gm
6.	Single shell weight	0.106 gm
7.	Single pupal weight	0.617 gm
8.	Silk ratio%	14.76%
9.	Shell weight after degumming	14.5 gm
10.	Spun yarn weight	9.8 gm
11.	Waste weight	4.7 gm
12.	Silk recovery%	67.59%
13.	Waste%	32.41%

The total cocoon weight was observed to be 105 gm. wherein total shell weight was 15.5 gm and total pupal weight was 89.5 gm. Single coccon weight, single shell weight and single pupal weight was 0.72 gm, 0.106 gm and 0.617 gm respectively. The silk ratio % was 14.76%. Shell weight after degumming was 14.5 gm. The Degumming loss (%) was found to be 6.45%. The spun yarn weight was found to be 9.8 gm. and silk waste was 4.7 gm. Thereby determining that the silk recovery % was 67.59% and Silk waste % was 32.41%.

## Discussion

### Exploring the insights of commercial utilization of *Cricula* silk

The cocoons were oval with tapering ends and contain perforation on the surface. When the cocoons were subjected to degumming the golden lusture was lost implying the affinity of the golden hue was high in the sericin content and not fibroin. Insights on developing an efficient degumming agent which aids in retaining the golden hue in the yarn may result in high demand of the fabric. The silk recovery % was observed to be higher than muga which was conventionally around 40–48% and higher yield was 55% in Muga silk.

*Cricula trifenestrata* fibre was reported for the tensile strength and biocompatibility. The modulus elasticity of the single fiber of *Cricula trifenestrata* is about 3681 MPa. The silk can be prepared by degumming method of boiling in 0.01 M NaOH for 1 h. Degumming above the concentration 0.01 M NaOH resulted in hydrolysis of fibre. The ultimate tensile strength is obtained about 162 MPa together with value of failure strain about 0.12 (Nindhia et al. 2014).

Unlike *Bombyx mori*, the pigments are usually deposited in the sericin which gets washed off during degumming process. However when cricula cocoons were degummed with chymotrypsin and the pigment content was observed in fibroin and sericin. It was observed that 1.4 times higher fold the pigment is present in fibroin than sericin (Yamada et al. 2001).

The wild silks in the world are still unexplored for their potential commercial utilization. Previous studies affirm the merits of *Cricula* silk for instance; *C. trifenestrata* silk protein was isolated and studied. The molecular mass of sericin and fibroin were 400 kDa and 350 kDa respectively, however fibroin when later underwent reduction the molecular mass was 180 kDa. The amino acid constituents resembled with higher serine content (Yamada and Tsubouchi 2001) The polar and non polar amino acid ratio in cricula sericin was about 69:31 (Manesa et al. 2020). *Cricula trifenestrata* Sericin has anti-proliferative activity in feline kidney cells and Sericin-induced apoptosis (Liu et al. 2016). *Cricula trifenestrata* cocoon extract when treated on human fibroblasts revealed non cytotoxicty (Sunarintyas et al. 2012).

**Fig. 6:**
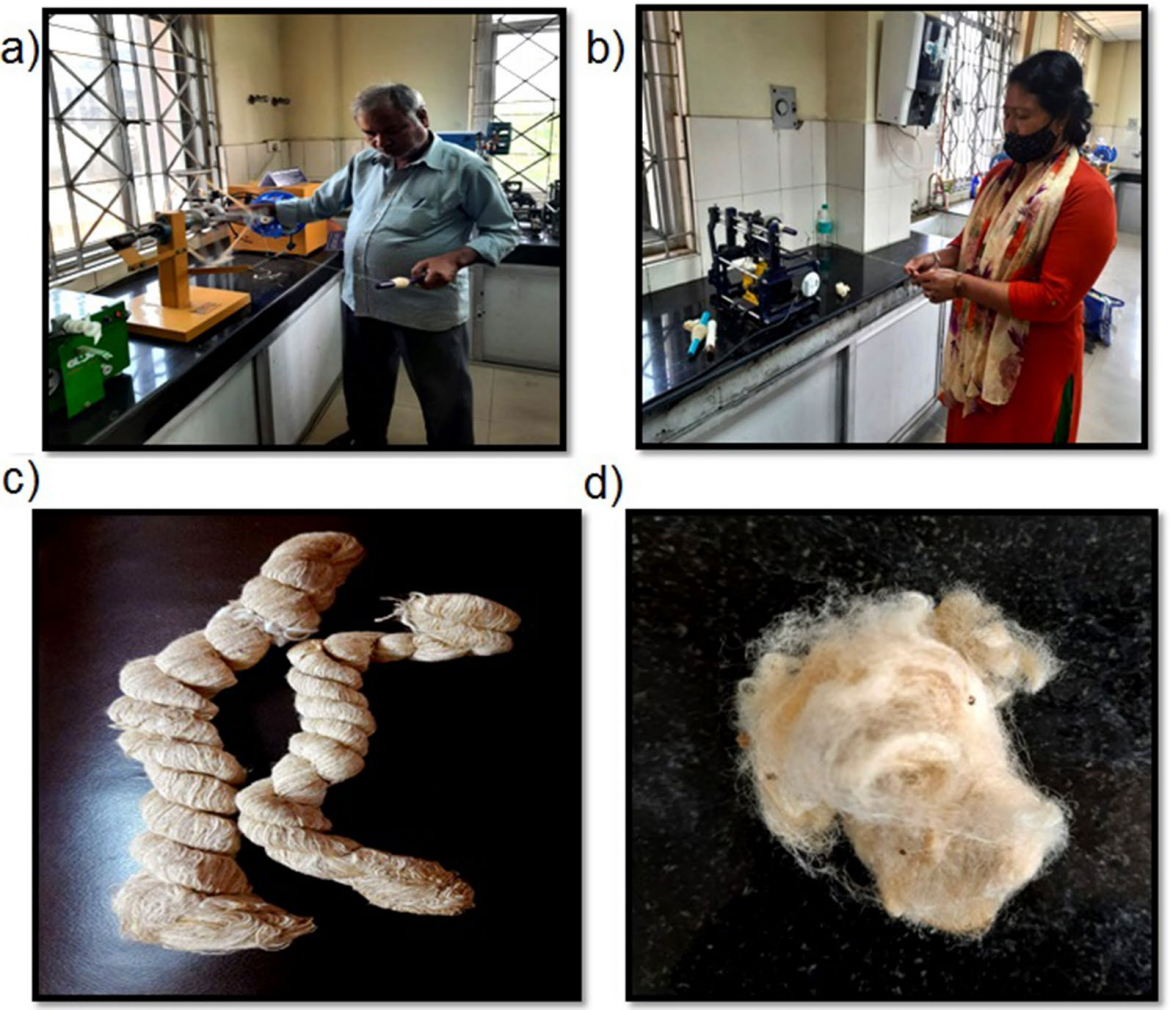
**a** Degummed cocoon, **b** the yarn is spun in motorized spinning machine, **c** Spun yarn hank preparation on approvate, **d** cricula spun yarn

This study affirms the efficacy of *Cricula* to be utilized as a spun silk (Fig. 6). The golden cocoons are utilized as artifacts for attractive aesthetic products.

## Conclusions

Popularization on the artistic utilization of these golden cocoons in fabric designing or any other products may be of high demand in the near future. In the current surge, *Cricula* sericin also has added biological effects in pharmaceuticals and cosmecutical industry. Hence more insights are to be explored for commercial product utilization of *Cricula* silk as a potential biomaterial, fabric, pharmaceutical, food, cosmetics and fuel industry.

## Data Availability

All data generated or analyzed during this study are included in this manuscript.

## Abbreviations

MT: Metric ton
NaOH: Sodium hydroxide
kDa: Kilo dalton
MPa: Mega pascal

## References

[CR1] Ahmed SA, Dutta LC, Sarmah MC (2012). Bio-efficacy of some insecticides against leaf eating caterpillar, *Cricula trifenestrata* Helfer (Lepidoptera: Saturniidae) Infesting Som, *Persea bombycina* Kost. Plantation Acad J Entomol.

[CR2] Alam MZ, Ahmed F (1992). Parasitoids associated with different stages of *Cricula trifenestrata* Helfer.

[CR3] Ali MI, Karim MA (1991). Notes on the biology, behaviour and biocontrol agents of mango defoliator *Cricula trifenestrata* (Lepidoptera: Saturniidae). Bangl J Entomol.

[CR4] Amin MR, Ahad MA, Rono MMA, Tithi DA (2008). Life history traits of *Cricula trifenestrata* (Lepidoptera: Saturniidae) feeding on Mangifera indica. J Agrofor Environ.

[CR5] Biswas S, Hath TK, Ray N (2013). Effect of different host plants on nutritional indices of wild silk moth. Cricula Trifenestrata J Entomol Res.

[CR6] Deshmukh PB (1992). Efficacy of two insecticide treatments against aphid, *Toxoptera avrantii*, and caterpillar, *Circula trifenesirata*, on groundnut, *Arachis hypogea* L. Int Pest Control.

[CR7] Gharde SK, Chaudhuri N (2018). The life history and population growth parameters of leaf eating caterpillar *Cricula trifenestrata* Helfer (Lepidoptera: Saturniidae) infesting manochilus bombycina king. Int J Curr Microbiol App Sci.

[CR8] Ghosh J, Chaudhuri N, Bera C (2015). Bio-ecological analysis to identify the critical stage of development of mango defoliator *Cricula trifenestrata* (Lepidoptera: Saturniidae). Int J Recent Sci Res.

[CR9] Huq SB, Hossain M, Khan AB (1991). Biology of *Cricula trifenestrata* (Lepidoptera: Saturniidae), a leaf eating caterpillar of mango. Bangl J Entomol.

[CR10] Kaleka AS, Singh D, Saini S (2018). Further studies on the moth *Cricula trifenestrata* from North-West India (Lepidoptera: Saturniidae). Ann Entomol.

[CR11] Liu W, Karimazawa M, Ozaki T, An Y, Miyazaki M, Suzuki K, Tsutsumi KI, Yamashita T (2016). Cell proliferation inhibition by sericin from the wild silkworm. Cricula Trifenestrata Adv Biol Chem.

[CR12] Manesa KC, Kebede TG, Dube S, Nindi MM (2020). Profiling of Silk Sericin from cocoons of three Southern African Wild silk moths with a focus on their antimicrobial and antioxidant properties. Materials.

[CR13] Munaan A (1986). Controlling *Cricula trifenestrata* Helf. on cashew trees. Pemberitaan Penelitian Tanaman Industri.

[CR14] Munshi R, Mazumdar S, Gupta PD, Chattopadhyay D (2016). Studies on standardization on degumming process for different eco races of eri silk cocoons. Indian J Nat Fibres.

[CR15] Narang RC, Gupta ML (1979). Chromosome number of *Cricula trifenestrata* Helfer (Lepidoptera: Saturniidae). Curr Sci.

[CR16] Negi BK, Barah A, Siddiqui AA, Sengupta AK (1993) *Cricula trifenestrata* (Lepidoptera: Saturniidae)-a new alternate host of Blepharipa zebina (Diptera: Tachinidae). In: Recent advances in Uzi fly research: proceedings of the national seminar on Uzi fly and its control, 16–17 January, 1992. Karnataka State Sericulture Development Institute, pp 269–271.

[CR17] Nindhia TGT, Knejzlik Z, Ruml T, Nindhia TS (2014). Tensile properties and biocompatibility of indonesian wild silk *Cricula trifenestrata*: a preliminary study. J Med Bioeng.

[CR18] Sarma AK, Gupta MK, Singh KM (2006). New record of a dipteran endoparasitoid of *Cricula trifenestrata* Helfer on Som *Machilus Bombycina*. J Plant Protect Environ.

[CR19] Singh SP (1992). Frequent outbreaks of *Cricula trifenestrata* Helfer (Lepidoptera, Saturniidae) on mango. Indian J Plant Protect.

[CR20] Siswanti R, Supriyadi S, Subagiya S (2017). Correlation between damage plant by silkworm *Cricula trifenestrata* to cashew yield. Agrotechnol Res J.

[CR21] Sjafaruddin M, Rahmatia D (1999) Field trial of *Beauveria bassiana* to canarium moth (*Cricula trifenestrata*) on cashew crop. In: Seminar Nasional Hasil Pengkajian dan Penelitian Teknologi Pertanian Menghadapi Era Otonomi Daerah, Palu (Indonesia), 3–4 Nov 1999. PSE.

[CR22] Solihin DD, Noor RR, Thohari AM (2012). The characteristics of cytochrome C oxidase gene subunit I in wild silkmoth *Cricula trifenestrata* helfer and its evaluation for species marker. Media Peternakan.

[CR23] Sunarintyas S, Siswomihardjo W, Tontowi AE (2012). Cytotoxicity of *Cricula triphenestrata* cocoon extract on human fibroblasts. Int J Biomater.

[CR24] Tikader A (2012). New record of Brachymeria tibialis (Walker)(Hymenoptera: Chalcididae) on *Cricula trifenestrata* (Helfer) from India. J Mun Ent Zool.

[CR25] Tikader A, Vijayan K, Saratchandra B (2014). *Cricula trifenestrata* (Helfer)(Lepidoptera: Saturniidae)-A silk producing wild insect in India. Trop Lepidoptera Res.

[CR26] UK C (2014) *Cricula trifenestrata* ((Helfer)), tea flush worm.[pest/pathogen]. *Cricula trifenestrata ((Helfer)), tea flush worm.[pest/pathogen].*, (AQB CPC record).

[CR27] Van Zwet AJ (1950). A practical method of cadavontrolling caterpillars in cinchona plantations. Bergcultures.

[CR28] Yadav S, Kumar A, Arvind K (2004). New record of wild silk caterpillar, cricula trifenestrata helfer on large cardamom and notes on it's biology. Advances in Life Sciences.

[CR29] Yamada H, Tsubouchi K (2001). Characterization of silk proteins in the cocoon fibers of *Cricula trifenestrata*. Int J Wild Silkmoth Silk (jpn).

[CR30] Yamada H, Kato Y, Tsubouchi K (2001). Yellow pigmentation of the fibroin core in the cocoon fibers of *Cricula trifenestrata*. Int J Wild Silkmoth Silk (jpn).

